# Psychometric properties of the Chinese version of the recovery locus of control (RLOC) Scale among patients with myocardial infarction

**DOI:** 10.1002/nop2.568

**Published:** 2020-07-23

**Authors:** Yuli Li, Dong Kong, Wei Wang, Yanhua Zhu, Xiaoqin Liu

**Affiliations:** ^1^ School of Nursing Cheeloo College of Medicine Shandong University Jinan China; ^2^ Nursing Department Shandong Provincial Hospital Shandong First Medical University Jinan China; ^3^ Department of Cardiology Shandong Provincial Hospital Shandong First Medical University Jinan China; ^4^ Medical Intensive Care Unit Shandong Provincial Hospital Shandong First Medical University Jinan China

**Keywords:** myocardial infarction, nurses, nursing, psychometric evaluation, recovery locus of control scale

## Abstract

**Aim:**

To translate the RLOC Scale into Chinese and test its psychometric properties in the Chinese patients with myocardial infarction (MI).

**Design:**

A cross‐sectional design was used.

**Methods:**

A convenience sample of 233 patients with MI who have undergone percutaneous coronary intervention and were ready for discharge were recruited in a level A tertiary hospital of Shandong Province from January 2019 to April 2019. Data were analysed using item analysis, internal consistency reliability and exploratory factor analysis.

**Results:**

Two factors—external RLOC and internal RLOC—were extracted, accounting for 70.5% of the variance. Cronbach's alpha for the Chinese version of RLOC Scale was 0.80 and for the two factors was 0.92 and 0.76, respectively. The Chinese version of RLOC Scale showed satisfactory reliability and validity, which can be used to measure the ability of recovery locus of control in Chinese patients with MI.

## 1 INTRODUCTION

Perceived control of recovery is an individual belief that patients have about their recovery process, which affects their involvement in health‐related behaviours considered to enhance the recovery process (Johnston, Morrison, Macwalter, & Partridge, 1999). Studies have shown that greater perceived personal control, that is an internal locus of control, is associated with more beneficial outcomes. For example, in the health filed, this result has been found for Parkinson's disease (Rizza et al., 2017), for medication adherence (Nafradi, Nakamoto, & Schulz, 2017) and for health‐promoting behaviours in Chinese patients with coronary heart disease (Zou, Tian, Chen, Cheng, & Fan, 2017).

Myocardial infarction (MI) is now a major cause of morbidity and mortality in China, with a rapid increase in the number of affected patients between 1999–2015 (Zhao et al., 2017) Cardiac remodelling and subsequent heart failure remain critical issues after MI despite improved treatment and reperfusion strategies (Haubner et al., 2016). The burden of MI remains high, currently causing one million deaths annually (Li et al., 2016). Therefore, a comprehensive strategy for secondary prevention is warranted among patients, after an initial MI, with the need for cardiac rehabilitation beginning soon after discharge from the hospital (Reed, Rossi, & Cannon, 2017). In the literature on coping and health, patients' self‐management, particularly their perception of the extent of control over processes and outcomes (in other words, perceived personal control), is shown to play an important role in enhancing recovery.

Recovery locus of control (RLOC) is a personality trait, which affects patients' involvement in health‐related behaviours, particularly self‐management (Partridge & Johnston, 1989; Thakral, Bhatia, Gettig, Nimgaonkar, & Deshpande, 2014). A previous study found that stroke patients who were able to positively perceive their level of self‐control and overcome negative thoughts towards stroke had faster recovery (Thompson, 1991). Stroke patients with a higher RLOC also had increased physical functioning (Mohd Zulkifly, Ghazali, Che Din, Desa, & Raymond, 2015). However, to our knowledge, there are no published papers about RLOC among patients with MI.

Various measures of health‐related locus of control exist. For example, Wallston, Wallston, Kaplan, and Maides, (1976) and Wallston, Wallston, and Devellis, (1978) Health Locus of Control (HLOC) Scale, which was later developed into the Multidimensional Health Locus of Control (MHLC) Scale, is widely used. Yet, they focus on preventive health behaviours make them unsuitable for measuring perceived control over existing severe physical disease. Partridge and Johnston (1989) developed a situation‐specific measure, the RLOC Scale, to predict the behaviour cognitions of individuals in the context of a physical disability (e.g. patients with stroke). Patients’ perceived control over the recovery process is presented as the RLOC, characterized by internal recovery locus of control (IRLOC) and external recovery locus of control (ERLOC); IRLOC is the patient's belief that their health condition depends on themselves, while ERLOC is the belief that it is determined by external environmental factors (Partridge & Johnston, 1989). Studies have shown that patients with higher IRLOC have faster recovery (Hanusch, O'Connor, Ions, Scott, & Gregg, 2014; Shaw, McColl, & Bond, 2003). The post‐MI recovery period can be a confusing, emotional time. However, MI patients’ behaviour cognitions of self‐care over the recovery process play an important role in improving outcomes, preventing hospital readmission and another MI. Thereby, it is important to choose a situation‐specific evaluation instrument to estimate patients’ recovery beliefs before health caregivers effectively empower patients and their families, and engage patients in self‐management and health behaviour change. Hence, the RLOC Scale is an ideal assessment tool to evaluate MI patients’ recovery locus of control.

The original version of the RLOC Scale, developed by Partridge and Johnston (1989), has been tested in European countries with good psychometric properties, in patients with physical disabilities or stroke. However, little is known about how the RLOC Scale performs in Chinese patients with MI. In fact, to date, no study examining the RLOC Scale in Chinese samples has been published in an English‐language journal. It is important for nurses to know the self‐efficacy regarding personal care in patients with myocardial infarction (MI), to identify individuals at risk and to make care plans. Assessment using the RLOC Scale and consequent treatment is expected to reduce psychological effects such as depression and loss of personal control due to MI, as it is believed to encourage patients to have positive perceptions towards their recovery. In addition to treatment, establishing an understanding of patients’ RLOC can not only reduce their psychological burden but also promote recovery from disease. Thus, patients can improve their physical functioning and avoid critical issues in recovery. The use of a reliable and valid instrument that measures the RLOC may stimulate further research related to health promotion and chronic disease self‐management. Therefore, the aim of this study was to test the validity and reliability of the Chinese version of the RLOC Scale among patients with MI.

## 2 METHODS

### 2.1 Study design and sample

This study used a cross‐sectional survey design. The participants were 285 patients with MI who had undergone percutaneous coronary intervention (PCI) and were ready for discharge. They were recruited from a level A tertiary hospital of Shandong Province between January and April 2019. Of them, 52 were excluded from the study for the following reasons: 28 patients were diagnosed by their doctor to have cognitive impairment; nine patients had auditory dysfunction and/or dyslexia; one patient had a malignant tumour; three patients had severe liver and kidney disease; and 11 patients refused to take part in this survey or were eliminated for other reasons. Finally, 233 patients were included in the study. The sample size was estimated based on the criterion for psychometric assessment of an instrument requiring 5–10 subjects per item (Nunnally & Bernstein, 1994). For the nine‐item Chinese version of the RLOC Scale, a sample of at least 90 subjects would be required; therefore, the sample size used in this study was reasonable.

Inclusion criteria were as follows: (a) patients with MI who underwent PCI and were ready for discharge; (b) aged between 18–75 years; and (c) able to read and speak Chinese. Exclusion criteria were as follows: (a) patients with malignant tumours, such as colorectal cancer, oesophageal cancer, gastric cancer or liver cancer; (b) severely impaired renal function (with estimated glomerular filtration rate < 30 ml/min/1.73 m^2^ or on dialysis); (c) female patients in the pregnancy or suckling period; (d) patients with auditory dysfunction and/or dyslexia; and (e) patients with cognitive impairment and/or mental disorders.

### 2.2 Ethical considerations and procedures

The study was approved by the ethics committee of Shandong Provincial Hospital affiliated to Shandong University (No. 2019‐073). The participants were informed of the purpose and procedure of the study and of their right to leave the study at any time or refrain from answering any questions. Written informed consent was obtained from all participants. The study was conducted in accordance with the tenets of the Declaration of Helsinki (World Medical Association, 2013). Eligible participants were referred by the nurses in the cardiovascular unit of the hospital. The nurses administered the Chinese version of the RLOC Scale and the Mini‐Mental State Examination (MMSE) and collected sociodemographic information, the day before their discharge. To determine whether they met the inclusion and exclusion criteria, the patients' clinical data were collected from their clinical cases files, with the permission of the patients and their cardiologists.

### 2.3 Instruments of data collection

The nine‐item RLOC Scale is a self‐report tool with five internal and four external items (Partridge & Johnston, 1989). IRLOC measures the belief that patients’ health condition depends on themselves, whereas ERLOC (4 items) measures the belief that patients’ health condition is determined by external environmental factors. Each item is rated on a five‐point Likert scale ranging from 1 (strongly agree) to 5 (strongly disagree), providing a total score in the range of 9–45, with a higher score indicating a better RLOC. The construct validity for the two‐factor model has been demonstrated, and the original scale has been shown to have internal consistency of, respectively, 0.50–0.77 (Johnston et al., 1999). The sociodemographic variables and clinical data obtained from the participants included sex, age, marital status, residence, educational level, occupation and the primary caregiver.

### 2.4 Translation procedures

The RLOC Scale was translated into Chinese using Brislin's (1986) forward and backward translation method. The translation of the RLOC Scale from the original English to Chinese was first performed by two independent and professional translators each (A and B) in the research team. The two translated versions (RLOC Scale‐A and RLOC Scale‐B) were merged into a single forward translation version (RLOC Scale‐C) by a third professional and native Chinese speaker (C). This version was then translated back into English by a fourth, bilingual researcher (D) who was not exposed to the scale previously. Discrepancies between the original and the back‐translated versions were reviewed for equivalence of meaning. Finally, the Chinese version of the RLOC Scale was modified and refined.

The translated RLOC Scale was pilot tested on a convenience sample of 20 patients with MI. Based on the test, problems concerning clarity, comprehension and interpretability were discussed. For example, the item “It is often best to just wait and see what happens” was difficult for patients to understand and we discussed how to express it more clearly and comprehensibly. The Chinese version of the RLOC Scale was finalized when no substantial disagreements remained, as shown in Table 1.

**Table 1:** The items in the Chinese version of the RLOC Scale

Item number	English items	Items translated into chinese
IRLOC items		
1	How I manage in the future depends on me not on what other people can do for me	未来如何管理疾病取决于我自己而不是取决于别人能为我做什么。
3	It's what I do to help myself that's really going make all the difference	自我帮助是实现疾病恢复的关键。
5	It's up to me to make sure I make the best recovery possible under the circumstances	依据目前的病情, 由我决定能否能获得最好地恢复。
7	Getting better now is a matter of my own determination rather than anything else	现在恢复疾病事关我的决心而不是其他东西。
9	It doesn't matter how much help you get‐ in the end it's your own efforts that count	疾病的恢复不在于你得到多少帮助, 而在于自己的努力。
ERLOC items		
2	It's often best to just wait and see what happens	通常对于疾病恢复进程最好是等等看。
4	My own efforts are not very important, my recovery really depends on others	我的疾病恢主要依赖别人, 而自己的努力并不重要。
6	My own contribution on my recovery doesn't amount to much	我对自身疾病恢复贡献不大。
8	I have little or no control over my progress from now on.	从现在起, 我较少控制或无法控制疾病恢复进程。

Abbreviations: ERLOC, external recovery locus of control; IRLOC, internal recovery locus of control; RLOC, recovery locus of control.

### 2.5 Data analysis

Statistical analyses were conducted using SPSS version 22.0. Significance levels were set at *p*‐value < .05. Descriptive statistics, including frequencies, percentages, means and standard deviations, were used to summarize the sociodemographic characteristics of the patients. The Shapiro–Wilk method was carried out to test the normality of the RLOC Scale data. The upper and lower 27% rule is commonly used in item analysis based on Kelley’s (1939) derivation, and any item with ≥70% of the patients choosing the same extreme response option was considered non‐discriminative (Juniper, Guyatt, & Jaeschke, 1996). Cronbach's alpha and correlated item‐to‐total correlation coefficients were calculated to determine the internal consistency of the Chinese version of RLOC Scale. Values >0.7 and 0.3 of the respective parameters indicate adequate internal consistency (Nunnally, 1978).

Validity for the Chinese version of the RLOC Scale was evaluated by exploratory factor analysis with Promax. We first conducted the Kaiser–Meyer–Olkin (KMO) test and Bartlett's test to determine whether there were statistically significant correlations among items to perform this analysis.

Additionally, Pearson's correlation coefficients were used to examine the correlations between total RLOC and its subscales. A series of *t* tests and one‐way ANOVA tests were conducted to examine the relationships between sociodemographic variables and the Chinese version of the RLOC Scale.

## 3 RESULTS

### 3.1 Sample characteristics

A total of 233 hospitalized patients with MI were recruited in this study. The mean age of the sample was 61.5 (*SD* = 12.0) years. Fifty‐seven per cent of them were male and 45.9% came from the city. The primary caregivers were mainly the spouses (48.1%) and children (45.9%). More characteristics of the subjects are presented in Table 2. There were no statistically significant relationships among the sample characteristics and the total score of the Chinese version of RLOC Scale (*p* > .05).

**Table 2:** Characteristics of the participants (*n* = 233)

	*n (%)*	RLOC total score		
		*M (SD)*	*t/F*	*P*
Sex				
Male	135 (57.94)	29.62 (7.94)	0.79	.43
Female	98 (42.06)	28.78 (8.22)		
Age, year				
≤40	11 (4.72)	30.45 (7.35)	1.08	.37
41–50	27 (11.59)	28.56 (8.03)		
51–60	70 (30.04)	27.81 (8.34)		
61–70	76 (32.62)	30.34 (8.34)		
>71	49 (21.03)	29.80 (7.27)		
Marital status				
Married	201 (86.27)	29.38 (8.07)	0.53	.60
Divorced/separate	32 (13.73)	28.56 (8.07)		
Residence				
City	107 (45.92)	29.45 (8.00)	0.48	.62
Sub‐rural	56 (24.03)	29.86 (8.26)		
Rural	70 (30.04)	28.51 (8.03)		
Educational level				
≤Primary	21 (9.01)	30.62 (6.90)	0.71	.49
Middle	111 (47.64)	29.59 (7.70)		
≥High	101 (43.35)	28.62 (8.65)		
Occupation				
Unemployed/retired	141 (60.52)	29.26 (8.15)	0.63	.53
Freelance work	49 (21.03)	30.16 (7.45)		
Regular work	38 (16.31)	28.21 (8.46)		
Primary caregiver				
Spouse	112 (48.07)	28.78 (8.50)	0.46	.71
Parents	7 (3.00)	27.57 (6.68)		
Children	107 (45.92)	29.80 (7.83)		
Other relatives	7 (3.00)	30.57 (5.50)		

Abbreviations: *M*, mean; RLOC, recovery locus of control; *SD*, standard deviation.

### 3.2 The item analysis

Results showed that there was a significant difference in RLOC total score between the upper 27% group and the lower 27% group (*t* = 26.72, *p* < .001). Table 3 lists the distribution of the responses for each item in the Chinese version of the RLOC Scale. None of the items had more than 70% of the patients choosing the same extreme response option. All items were therefore considered discriminative.

**Table 3:** Item‐total correlations and distribution of the item responses (%)

	Skewness	Corrected item‐total correlation	Distribution of the item responses (%)				
			Strongly agree	Agree	Uncertain	Disagree	Strongly disagree
Internal items							
1	−0.32	0.80	6.4	18.9	8.2	40.3	26.2
3	−0.41	0.77	6.9	12.4	15.5	37.8	27.5
5	−0.35	0.80	4.7	16.7	20.6	39.1	18.9
7	−0.57	0.90	3.9	21	12.9	38.2	24
9	−0.47	0.73	3.4	23.6	16.3	35.6	21
External items							
2	−0.15	0.45	12	39.9	17.2	26.6	4.3
4	−0.27	0.70	6.4	49.8	15.5	22.3	6
6	−0.23	0.67	3.4	44.2	23.2	22.7	6.4
8	−0.08	0.43	1.3	32.2	39.5	21.9	5.2

### 3.3 Reliability

The internal consistency coefficient (Cronbach's alpha) for the Chinese version of RLOC Scale was 0.80 and for the two subscales was 0.92 and 0.76, respectively, which demonstrated all subscales had satisfactory internal consistency reliability (DeVellis, 2017). All items were above the level of 0.40, which indicated the item's homogeneity in measuring the concept of recovery locus of control (Table 3). The skewness value for all items ranged from −0.08 to −0.57. The Shapiro–Wilk normality statistic for the total score of the Chinese version of RLOC Scale was 0.99 (*p* = .25), and the total score of the Chinese version of the RLOC Scale and the subscale scores were significantly correlated (*p* < .01).

### 3.4 Validity

The exploratory factor analysis showed that the KMO measure was 0.82 and the approximate chi‐square value for Bartlett's test was 1,190.61 (*df* = 36, *p* < .001). Then, the unweighted least squares (ULS) was used to extract factors and the rotation method Promax was used, as the two factors were significantly correlated. The two factors explained a total of 70.50% of the total variance, and the per cent variances for the two factors were 47.82% and 22.68%, respectively, and were extracted with eigenvalues > 1. The factor loadings of all nine items ranged from 0.58 (item 8) to 0.94 (item 7), as shown in Table 4.

**Table 4:** Factor analysis results for the Chinese version of the RLOC Scale (*n* = 233)

Item number	Pattern matrix		Structure matrix	
	Factor 1	Factor 2	Factor 1	Factor 2
1	0.80		0.80	
3	0.76		0.81	
5	0.91		0.88	
7	0.91		0.88	
9	0.66		0.66	
2		0.64		0.65
4		0.82		0.82
6		0.77		0.78
8		0.55		0.52
Eigenvalue	5.35	2.54		
Per cent variance (%)	47.82	22.68		

Abbreviation: RLOC, recovery locus of control.

## 4 DISCUSSION

The purpose of the study was to examine the psychometric properties of the Chinese version of the RLOC Scale among hospitalized patients with myocardial infarction. The scale was originally constructed to fit the context of rehabilitation and recovery from physical disability, such as hemiplegia resulting from a stroke, in the Western culture. In this study, the Chinese version of the RLOC Scale was tested, with acceptable reliability and validity for use among hospitalized patients with myocardial infarction, thereby expanding the range of application of the original scale.

In this study, the item analysis demonstrated the Chinese version of the RLOC Scale was reliable. Cronbach's alpha coefficient was 0.80 for the total scale, 0.92 and 0.76 for the two factors respectively, which were >0.70 indicated adequate internal consistency (Nunnally, 1978). These results were better than those found in a previous study with stroke patients (Johnston et al., 1999). By using exploratory factor analysis, all items were found to load on two distinctive factors: IRLOC and ERLOC, as proposed for the original version (Partridge & Johnston, 1989). These results demonstrated that the RLOC was validated in post‐MI populations other than stroke patients. The Chinese version of RLOC Scale will be useful instruments to estimate post‐MI patients’ recovery beliefs, which, in turn, could help health caregivers provide strategies to build self‐confidence in the recovery process and engage patients in self‐management. Additionally, the Chinese version of RLOC will help researchers to conduct quantitative study to explore factors related to post‐MI patients’ recovery locus and provide more specific interventions.

Though the Chinese version of the RLOC Scale proved to be a reliable and valid instrument in assessing the RLOC in Chinese patients with MI, there were limitations in the current study. First, recruiting a convenience sample of patients with MI from a level A tertiary hospital may limit the generalizability of the findings to patients in the county hospitals. Moreover, there were no statistically significant relationships among the sample characteristics and the total score of the Chinese version of the RLOC Scale, which might be explained by sampling error. Second, the cross‐sectional data collection design and the recruitment of patients soon to be discharged did not allow for the evaluation of the test–retest reliability. Finally, the face validity and the convergent validity of the Chinese version of RLOC Scale were not tested in the current study, which may limit the estimates of validity. In the future, the convergent validity of the Chinese version of the RLOC Scale could be tested by comparing the scale to other self‐efficacy or RLOC scales.

## 5 CONCLUSIONS

The Chinese version of RLOC Scale had satisfactory reliability and validity and can be used to measure the RLOC in Chinese patients with MI. This opens up opportunities for further research to develop interventions aiming to improve the RLOC or self‐efficacy regarding personal care in patients with MI, with the aim of increasing their involvement in health‐related behaviours designed to enhance recovery process, especially in the personal care context.

## AUTHOR CONTRIBUTIONS

YL. L and YH. Z: Study design. DK, WW and YH. Z: Data collection. YL. L and XQ. L: Data analysis. YL. L, DK, WW, YH. Z and XQ. L: Manuscript writing and revisions for important intellectual content.

## ETHICAL APPROVAL

Written informed consent was obtained from all participants, and Institutional Review Board approval (No.2019‐073) was obtained from Shandong Provincial Hospital affiliated to Shandong University.
